# Supplementary material for: Genetics of nodulation in Aeschynomene evenia uncovers mechanisms of the rhizobium–legume symbiosis
Source: Nat Commun. 2021 Feb 5;12:829. doi: 10.1038/s41467-021-21094-7 (PMC7864950; doi:10.1038/s41467-021-21094-7)
Supplement: Supplementary file 3 — Reporting Summary [file 41467_2021_21094_MOESM3_ESM.pdf]

nature research

Corresponding author(s): Jean-François Arrighi

Last updated by author(s): 2020 12 21

Reporting Summary

Nature Research wishes to improve the reproducibility of the work that we publish. This form provides structure for consistency and transparency in reporting. For further information on Nature Research policies, see our Editorial Policies and the Editorial Policy Checklist.

Statistics

For all statistical analyses, confirm that the following items are present in the figure legend, table legend, main text, or Methods section.

n/a	Confirmed	
☐	☒	The exact sample size (*n*) for each experimental group/condition, given as a discrete number and unit of measurement
☐	☒	A statement on whether measurements were taken from distinct samples or whether the same sample was measured repeatedly
☒	☐	The statistical test(s) used AND whether they are one- or two-sided<br>*Only common tests should be described solely by name; describe more complex techniques in the Methods section.*
☒	☐	A description of all covariates tested
☒	☐	A description of any assumptions or corrections, such as tests of normality and adjustment for multiple comparisons
☐	☒	A full description of the statistical parameters including central tendency (e.g. means) or other basic estimates (e.g. regression coefficient) AND variation (e.g. standard deviation) or associated estimates of uncertainty (e.g. confidence intervals)
☐	☒	For null hypothesis testing, the test statistic (e.g. *F*, *t*, *r*) with confidence intervals, effect sizes, degrees of freedom and *P* value noted<br>*Give P values as exact values whenever suitable.*
☐	☒	For Bayesian analysis, information on the choice of priors and Markov chain Monte Carlo settings
☒	☐	For hierarchical and complex designs, identification of the appropriate level for tests and full reporting of outcomes
☒	☐	Estimates of effect sizes (e.g. Cohen's *d*, Pearson's *r*), indicating how they were calculated

Our web collection on statistics for biologists contains articles on many of the points above.

Software and code

Policy information about availability of computer code

Data collection	No software used for collecting data
Data analysis	Detailed description for all the softwares used for analysis have been provided in the Methods as well as Supplementary Information. The tools and softwares used in this study: HGAP, PbcR-MHAP (version wgs-8.3rc2), Falcon (v2.3), the wgs-assembler software package (former CELERA), Quiver algorithm (SMRT Analysis v2.3.0), SSPACE-LongRead (v1.1), SPADES, GenoScope v1, Jellyfish v2.1.1, Bowtie2, Tassel (v5.0), JoinMap v4, Scaffhunter, GBS_corrector.0.3.py, SpiderMap v1.6.12b, GenoGraph v2.2, Megablast, TrimGalore v0.6.0, SMRT Analysis (v2.2), Quiver, RepeatModeler v1.0.11, RepeatMasker v4-0-7, STAR v2.7, StringTie v1.3.3b, Cuffmerge from Cufflinks v2.2.1 , TopHat v2.0.14 , AUGUSTUS v 3.2.3, EVM v1.1.1, TransDecoder v2.1.0, BUSCO v3, BLASP, BLAST, InterProScan, AHRD, INFERNALv1.1.2, RNAmmer v1.2, tRNAscan-SE v1.3.1, RepeatModeler v1.0.11, Tandem Repeat Finder v 4.04, SynMap (CoGe), NGPhylogeny.fr (https://ngphylogeny.fr/), Circos v0.69, JCVI, bwa mem v0.7.12-r1039, samtools v1.3.1, freebayes v 0.9.7, IQ-TREE, iTOL v 4.3, RSEM v1.3.0, DRAP, OrthoFinder v0.4.0, MAFFT v7.205, FastTree v2.1.5, FigTree v1.4.3, Genome Viewer Context, (Legume Information System), GEvo (CoGe), Muscle v 0.1, MrBayes (v 3.2.2), Multalin, Expasy, Jalview v2.11.0, FastQC, SnpEff v4.3t, PhyML v3.1_1 , PoolSeq v0.3.3, WebLogo v3.7.

For manuscripts utilizing custom algorithms or software that are central to the research but not yet described in published literature, software must be made available to editors and reviewers. We strongly encourage code deposition in a community repository (e.g. GitHub). See the Nature Research guidelines for submitting code & software for further information.

Data

Policy information about availability of data

All manuscripts must include a data availability statement. This statement should provide the following information, where applicable:

- Accession codes, unique identifiers, or web links for publicly available datasets
- A list of figures that have associated raw data
- A description of any restrictions on data availability

The data reported in this study are tabulated in Datasets S1–S9 and SI Appendix. Genome assembly and annotation, accession resequencing and RNA-seq data for A. evenia are deposited at NCBI under BioProject ID: PRJNA448804. RNA-seq data for other Aeschynomene species are available under the BioProject ID: PRJNA459484. Resequencing data for A. evenia nodulation mutants are available under the BioProject ID: PRJNA590707 and PRJNA590847. Accession numbers for all deposited data are given in Supplementary Dataset 9. Genome assembly and annotation for A. evenia can also be accessed at the AeschynomeneBase (http://aeschynomenebase.fr) and at the Legume Information System (https://legumeinfo.org). Additional data were obtained from the SwissProt database (https://www.uniprot.org), InterPro (https://www.ebi.ac.uk/interpro/), GO (http://geneontology.org/), the KEGG pathways database (https://www.genome.jp/kegg/pathway.html), Legume Mines (https://mines.legumeinfo.org) and CoGe (https://genomevolution.org). Biological material and constructs are available for academic research upon reasonable request.

Field-specific reporting

Please select the one below that is the best fit for your research. If you are not sure, read the appropriate sections before making your selection.

☒ Life sciences ☐ Behavioural & social sciences ☐ Ecological, evolutionary & environmental sciences

For a reference copy of the document with all sections, see nature.com/documents/nr-reporting-summary-flat.pdf

Life sciences study design

All studies must disclose on these points even when the disclosure is negative.

Sample size	No statistical methods were used to predetermine sample sizes. For RNAseq analyses, biological material was collected from at least six plants per condition and pooled before RNA extraction. For cytometry analysis, DNA content measurement was performed separately on material from three plants per condition, with at least 10,000 stained isolated nuclei analysed per sample. For the genetic analysis of nodulation mutants, a large number of analyzed plants was used to ensure enough sequencing coverage and data robustness (use of 300 to 600 F2 plants for the genetic determinism analysis, use of 20 F2 mutant plants for the genotyping analysis, several crosses performed with each mutant for the allelism tests).
Data exclusions	No data was excluded from the analysis
Replication	For the mutant analysis, nodulation phenotypes were obtained over a period of 4 years. They were shown to be constant over the different experiments, over plant generations and between allelic mutants. In total, for each mutant, root phenotypes were observed four times and stem phenotypes at least twice .
Randomization	To generate biological material and for the mutant phenotype analysis, different lines were cultured in the same containers either in the greenhouse or in the growth chamber, so as to guarantee identifical growth conditions.
Blinding	Mutant phenotypes were initially characterized before identifying mutation in symbiotic genes. Flow cytometry data were obtained and analyzed by the cytometry plaform without knowledge of the in silico symbiotic gene analysis.

Reporting for specific materials, systems and methods

We require information from authors about some types of materials, experimental systems and methods used in many studies. Here, indicate whether each material, system or method listed is relevant to your study. If you are not sure if a list item applies to your research, read the appropriate section before selecting a response.

Materials & experimental systems

n/a	Involved in the study	
☒	☐	Antibodies
☒	☐	Eukaryotic cell lines
☒	☐	Palaeontology and archaeology
☒	☐	Animals and other organisms
☒	☐	Human research participants
☒	☐	Clinical data
☒	☐	Dual use research of concern

Methods

n/a	Involved in the study	
☒	☐	ChIP-seq
☐	☒	Flow cytometry
☒	☐	MRI-based neuroimaging

Flow Cytometry

Plots

Confirm that:

- [ ] The axis labels state the marker and fluorochrome used (e.g. CD4-FITC).
- [ ] The axis scales are clearly visible. Include numbers along axes only for bottom left plot of group (a 'group' is an analysis of identical markers).
- [ ] All plots are contour plots with outliers or pseudocolor plots.
- [ ] A numerical value for number of cells or percentage (with statistics) is provided.

Methodology

Sample preparation	Nuclei were isolated from roots and nodules and analysed as indicated in the Supplementary Notes
Instrument	Cytometer CytoFLEX S, Beckman Coulter.
Software	*Describe the software used to collect and analyze the flow cytometry data. For custom code that has been deposited into a community repository, provide accession details.*
Cell population abundance	Endopolyploidy of at least 10,000 stained isolated nuclei was determined for each sample.
Gating strategy	*Describe the gating strategy used for all relevant experiments, specifying the preliminary FSC/SSC gates of the starting cell population, indicating where boundaries between "positive" and "negative" staining cell populations are defined.*

- [ ] Tick this box to confirm that a figure exemplifying the gating strategy is provided in the Supplementary Information.
