# Supplementary material for: Genetics of nodulation in Aeschynomene evenia uncovers mechanisms of the rhizobium–legume symbiosis
Source: Nat Commun. 2021 Feb 5;12:829. doi: 10.1038/s41467-021-21094-7 (PMC7864950; doi:10.1038/s41467-021-21094-7)
Supplement: Supplementary file 4 — Description of Additional Supplementary Files [file 41467_2021_21094_MOESM4_ESM.pdf]

Description of Additional Supplementary Files

Supplementary Data 1. Summary of intrasyntenic blocks found in the Aeschynomene evenia genome

Supplementary Data 2. Summary of synteny blocks between Aeschynomene evenia and Arachis duranensis

Supplementary Data 3. Summary of synteny blocks between Aeschynomene evenia and Arachis ipaiensis

Supplementary Data 4. The LysM-RLK/RLP gene family in A. evenia and comparison with other legume species

Supplementary Data 5: List of symbiotic genes investigated in A. evenia and comparison with other legume species.

Supplementary Data 6. Nodule-specific Cys-rich genes from A. evenia. The NCR genes were named with the initial of the plant species and numbered according to their position in the A. evenia genome. NCR motifs 1 and 2 correspond to those deduced from sequence alignements in Supplementary Figure 7. Theorical pI was calculated for mature peptides. Tissue expressions are given for the two A. evenia accessions CIAT22838 and IRFL6945.
