# Supplementary material for: Genetics of nodulation in Aeschynomene evenia uncovers mechanisms of the rhizobium–legume symbiosis
Source: Nat Commun. 2021 Feb 5;12:829. doi: 10.1038/s41467-021-21094-7 (PMC7864950; doi:10.1038/s41467-021-21094-7)
Supplement: Supplementary file 2 — Peer Review File [file 41467_2021_21094_MOESM2_ESM.pdf]

Reviewers' comments:

Reviewer #1 (Remarks to the Author):

In this manuscript entitled “genome of the tropical legume Aeschynomene evenia reveals new mechanisms of the nitrogen-fixing rhizobium symbiosis” the authors report i) the first genome assembly for a nodulation Nod-factor independent Aeschynomene species, ii) the description of the evolution of candidate symbiotic genes and iii) a forward genetic screen leading to the discovery of a novel symbiotic gene, AeCRK, potentially specific of Nod-factor independent Aeschynomene.

I found the presented work very impressive and exciting. It goes beyond the “usual” genome paper by including phylogenetic analyses but also biochemical evidence (for the convergent recruitment of Leghemoglobin) and an ambitious forward genetic screen.

I have two requests that I think should be taken into account:

1) The evolution of AeCRK should be better analyzed. From the synteny analysis it seems that it originates from a tandem duplication. Figure 8f suggests that this symbiotic copy is present in Arachis too (and seems up-regulated in nodules according to LegumeMines). The authors should run phylogenies on a larger sampling of legume genomes to determine whether this copy is specific to the Dalbergoids or more broadly distributed. Its presence in Arachis clearly disconnects its evolution from the acquisition of NF-independent nodulation. Could it be linked to intercellular infection? Is it conserved in Papilionoids that have lost nodulation? The fact that mutants for the paralogs in Medicago or Lotus have never been identified by forward genetics may reflect functional redundancy within the cluster or a function not linked to nodulation. Could the author look at dN/dS on the Aeschynomene CRK to search for potential signature of neofunctionalization? The authors should expand by a few sentence the current discussion to reflect the different possible scenarios.

2) Some of the results should be more discussed. The study includes many exciting results that are only shortly discussed. In term of evolutionary processes, the authors could discuss the diversification of Leghemoglobin (same function, different genes). The short parallel with Parasponia could be expanded. More importantly, the authors could elaborate on the impact of their work on deciphering NF-independent nodulation. Does it address some of the current questions? Finding that the entire CSP is present and seems required for nodulation implies that the difference may only come from the receptor plugged-in the pathway. Why are NFP/LYK3 still present (function in AM symbiosis? Other role?).

Minor comments.
- P7: WDG should be WGD
- Reference should be included for the material and method section on Globin spectral analyses

Pierre-Marc Delaux

Reviewer #2 (Remarks to the Author):

The authors de novo assembled a genome for Aeschynomene evenia, which has a unique symbiosis with photosynthetic bradyrhizobia that is Nod factor-independent process. Based on this genome, they did transcriptomic-based assembly for 10 additional Aeschynomene spp. and performed comparative analyses with other legume species that with Nod factor-dependent process. They found numbers of genes that may miss from the Aeschynomene spp. such as LYK3, EPR3, VAG1, SUNERGOS1, BIN3, BIN4…, Also, they screened EMS mutants and identified some previous reported genes and CRK.
It is an interesting topic, however, the analyses are not solid enough to support the findings. Here are some comments:

1. Given the high level of sequencing technology, it is easier to get a high quality reference genome now. However, with a scaffold N50 of ~0.985 Mb, the genome assembly of this study is poor. Moreover, only about 302 Mb (80%) of the total length of the assembly were anchored to the chromosomal pseudomolecules. I suggest the authors spend more effort to improve the genome quality since it is not a challenge now. Many approach would help, such as Hi-C and BioNano.

2. For the de novo RNA-seq assemblies, how about the linage of the annotation genes from RNA-seq to the annotation of this genome? How many orotholog genes could be identified in the RNA-seq assemblies comparing with the reference genome? The distribution of the gene (coding) lengths? Are they comparable to that from reference genome？

3. For the part of orotholog/comparative analyses part, the procedure is fine, which has been adopted in a number of studies. The authors found some genes may miss from the Aeschynomene spp. like LYK3, EPR3, VAG1, SUNERGOS1, BIN3, BIN4… I would like the authors to add more species, even from species of non-legume to further check if the missing of these genes were indeed associated with Nod factor-dependent/independent. Because missing gene is normal, but if it associate with a specific process is hard to determine, unless clear functional characterization. Now add more species at least to eliminate false judgment.

4. One more, based on the data, it is hard to see that NFP was not detected in all Aeschynomene transcriptomes (Fig. 3a).

5. The EMS database is interesting and would be helpful for further functional analyses. They found several reported genes: AePOLLUX, AeCCaMK, AeCYCLOPS, AeNSP2 and AeNIN. These genes also important for Nod factor-dependent process or even some closely related to Nod factor, does that mean Nod factor-independent process and Nod factor-dependent process still shared common pathway? Or another explanation?

6. The author found that CRK might be a good candidate for the Nod factor-independent process. Because here is a gene cluster, it might be a little bit complicate. Again, using more species (including species of more legume, non-legume, the10 additional Aeschynomene spp, and even the 12 additional resequenced A. evenia accessions) to check the phylogenetic relationship (instead of current Figure 8e), what is the results could be? I suggest to do more detail analyses in this part. It may make the data clear.

7. One more, the authors find a number of genes that may lost from the Aeschynomene spp. It is interesting that from the comparative, did the authors find any gain genes (beside the additional genes from gene cluster)? If so, when do the mutant screening, if these genes got mutation, does there any phenotype?

Reviewer #3 (Remarks to the Author):

A long-standing objective in legume-rhizobium research is to unravel the molecular genetic pathways controlling the nitrogen-fixing nodule symbiosis, in such that this knowledge can be used to engineer this trait in non-leguminous crops. Nitrogen-fixing nodule symbiosis is not unique to legumes, though current knowledge on the symbiotic gene networks is largely based on studies on two legume species of a single taxonomic clade; Medicago truncatula and Lotus japonicus (both belonging to the Hologalegina clade of the Papilionoideae subfamily). Two decades of research on these models has provided insights into the genetic pathways essential for establishing this symbiosis. Also, it was shown that these pathways are highly conserved in both species. Despite this substantial body of knowledge, the evolutionary basis of the nitrogen-fixing nodule symbiosis has not been uncovered. Only recently phylogenomic studies using nodulating non-model species provided some insights, indicating the recruitment of three genes to act specifically in the nitrogen-fixing nodule symbiosis. (i)

The LysM-type receptor NFP/NFR5 to perceive lipo-chitooligosaccharide signal molecules (known as Nod factors) secreted by the nitrogen-fixing microsymbiont, (ii) the symbiosis responsive transcription factor NIN that is essential -and sufficient- to trigger nodule organogenesis, and (iii) the coiled-coil protein RPG controlling the bacterial infection process.

The Arrighi team now presents a highly relevant system that can be used to obtain novel insights in an alternative evolutionary trajectory of the nitrogen-fixing symbiosis trait; Aeschynomene evenia. A. evenia represents a different taxonomic clade when compared to Medicago / Lotus (Dalbergioids, Papilionoideae). Dalbergioid species have a distinct infection mechanism when compared to most other legumes, not making use of root hair curling-based infection to guide rhizobium to the nodule primordium. Instead, rhizobia penetrate intercellularly via a mechanism known as crack entry. However, the most striking feature of A. evenia is that it is nodulated by rhizobia that do produce the canonical Nod factor signal molecules.

There are two aspects that make this manuscript highly relevant.
(1). The Arrighi team not only has generated a high-quality genome sequence of A. evenia (plus draft genome sequences of 12 additional accessions) but also has integrated it with the genetic map. This facilitates forward genetic screens, as is demonstrated in the manuscript to obtain insights in the molecular genetic mechanisms controlling Nod factor-independent nodulation. This achievement is unique in nodulation research, where forward genetic screens were limited to Medicago and Lotus, and occasionally a legume crop. Therefore, A. evenia can be considered as a third legume model species amenable for genetic research.
(2). By comparative analysis in combination with a close to saturating forward genetic screen, the core symbiosis network for Nod factor-independent nodulation has been identified. A whole set of genes known to be important for nodulation in Medicago and/or Lotus, showed not to be essential for nodulation of A. evenia; including LysM-type Nod factor receptors and the interacting flotillin (FLOT) protein, the LysM-type rhizobium exopolysaccharide receptor EPR3, the chitinase-like enzyme encoding gene NOD FACTOR HYDORLOASE1, RPG, and the genes controlling endoreplication in nodule cells VAG1, SUNERGOS1, BIN3 and BIN4. Additionally, by forward genetics a novel Cysteine-Rich receptor-kinase (AeCRK) was found to be essential for nodule formation in A. evenia. Taken together, I consider these insights highly relevant in the understanding of alternative molecular mechanisms that can lead to nodulation. This knowledge will be instrumental in engineering approaches.

Major issues:
On page 4 it is stated that A. evenia possesses the Nod factor receptor gene NFP, but that this gene is not expressed in a nodulation context. However, when I evaluate the phylogenetic tree presented in Fig. 3B, I do not find a support that A. evenia indeed has a gene orthologous to Medicago MtNFP and Lotus LjNFR5. The closest A. evenia homolog, Ae05g05580, is grouping with MtLYR1/LjLYS11. MtNFP/LjNFR5 and MtLYR1/LjLYS11 evolved most probable upon a Papilionoid-specific whole-genome duplication (see: Young et al., 2011 Nature). MtNFP/LjNFR5 is essential for rhizobium Nod factor-induced nodulation in Medicago and Lotus, however, no evidence has been found that MtLYR1/LjLYS11 plays a role in nodulation as well. Fig. 3B further indicates that also Arachis duranensis lacks a MtNFP/LjNFR5 ortholog, as Aradu.AXP1M also is more closely related to MtLYR1/LjLYS11 than to MtNFP/LjNFR5. These issues have to be resolved. Please, provide irrefutable evidence that A. evenia (and Arachis species) possesses an MtNFP/LjNFR5 orthologous gene (e.g. by phylogenetic analysis and protein alignments, genomic syntheny conservation, etc).

No complementation has been conducted for AeCRK, and I am wondering why, as Agrobacterium rhizogenes mediated transformation has been established for A. evenia (Arrighi et al 2012 MPMI). To my opinion, complementation of the Aecrk mutant is an omission.

It is reported that Aecrk mutants are nod minus, however, no detailed phenotypic analysis is presented. So, can it be confirmed that nodule organogenesis is fully blocked in Aecrk mutant plants? And what about rhizobium-induced NIN expression? Some basic phenotypic characterization is

welcomed.

Gene loss in A. evenia is described for EPR3, NFH1, RPG, FLOT1, VAG1, SUNERGOS1, BIN3, BIN4. However, the underlying data are not always obvious. Are the genes not annotated, pseudogenezed, completely eroded/deleted? Please, provide the data (as supplemental information) that unambiguously support the absence of functional copies of these genes.

page 8: Provide a list -supported by phylogenetic analysis- which LysM-RLK genes are absent in A. evenia.

Minor issues:
The loss of RPG is not unique to Aeschynomene, but also has been lost in Arachis. As RPG (like NFP) is considered to be one of the three genes that specifically evolved allowing nodulation, this finding should be addressed in the DISCUSSION section of the manuscript.

As several mutant alleles for POLLUX, CCAMK, CYCLOPS, NSP2 and NIN have been found, I consider your screen for nod minus mutants close to saturation. To my surprise, no NSP1 mutants have been found, whereas there are no obvious paralogs reported that may function redundantly (like is the case for SYMRK). This should be addressed in the DISCUSSION section of the manuscript.

page 4: "Previous transcriptome analysis did not detect the expression of several known genes involved in bacterial recognition, infection and nodule functioning". Provide information on which genes have been studied previously, and what tissues have been used for this.

page 6: Provide web address for AeschynomeneBase.

page 7, last paragraph. Fig. 3A is only a schematic representation of the gene species tree. Please, provide a high-resolution consensus species tree based on the inference of single copy orthogroups.

page 15, DISCUSSION, first paragraph. Information on "T3SS-mediated hijacking of the symbiotic signalling pathway" and " the absence of the T3SS in A. evenia compatible photosynthetic Bradyrhizobium species" has to be included in the INTRODUCTION of the manuscript.

Fig. 3A: The use of a single letter genus abbreviation for Arachis and Aeschynomene is confusing. Can be solved by using either two letters (Ar and Ae) or a colour indication of Aeschynonomene.

Fig. 3A and Fig. 4A can be merged. Fig. 4B could go to the supplement.

Fig. 3C: Include also EPR. To create space, Fig. 3D can go to the supplement.

Fig. 8f: Provide gene IDs of CRK genes (black arrows) similar as in Fig. 6d (LB genes).

Suppl. Fig. 4a, Supple Fig. 5a-b: Provide gene IDs of LYK, SYMRK, and PUB1 genes (black arrows in figures) similar as in Fig. 6d (LB genes).

Suppl. Fig. 4b-c, Suppl. Fig. 5c, Suppl. Fig. 6a-d: I find these screenshots messy and not easy to interpret. Please, replace them by schematic drawings (e.g. as in Suppl. Fig. 4a and Suppl. Fig. 5a-b) and provide all the alignments in a separate supplemental file.

Reviewers' comments:

We thank the reviewers for their appreciation of our work and for their constructive comments. They were very useful to improve the quality of our manuscript.

Reviewer #1 (Remarks to the Author):

In this manuscript entitled "genome of the tropical legume Aeschynomene evenia reveals new mechanisms of the nitrogen-fixing rhizobium symbiosis" the authors report i) the first genome assembly for a nodulation Nod-factor independent Aeschynomene species, ii) the description of the evolution of candidate symbiotic genes and iii) a forward genetic screen leading to the discovery of a novel symbiotic gene, AeCRK, potentially specific of Nod-factor independent Aeschynomene.

I found the presented work very impressive and exciting. It goes beyond the "usual" genome paper by including phylogenetic analyses but also biochemical evidence (for the convergent recruitment of Leghemoglobin) and an ambitious forward genetic screen.

I have two requests that I think should be taken into account:

1) **The evolution of AeCRK should be better analyzed. From the synteny analysis it seems that it originates from a tandem duplication. Figure 8f suggests that this symbiotic copy is present in Arachis too (and seems up-regulated in nodules according to LegumeMines). The authors should run phylogenies on a larger sampling of legume genomes to determine whether this copy is specific to the Dalbergoids or more broadly distributed. Its presence in Arachis clearly disconnects its evolution from the acquisition of NF-independent nodulation. Could it be linked to intercellular infection? Is it conserved in Papilionoids that have lost nodulation? The fact that mutants for the paralogs in Medicago or Lotus have never been identified by forward genetics may reflect functional redundancy within the cluster or a function not linked to nodulation. Could the author look at dN/dS on the Aeschynomene CRK to search for potential signature of neofunctionalization? The authors should expand by a few sentence the current discussion to reflect the different possible scenarios.**

1. As suggested by Reviewer #1, we expanded our phylogenetic analysis on a larger sampling of legumes by including additional Papilionoid species along with the more basal *Chamaecrista fasciculata* and *Mimosa pudica*. The observed distribution of AeCRK orthologs is linked to intercellular infection as pointed out by Reviewer #1and this observation is mentionned in the results:

L330: "Although similar CRK clusters are located in syntenic regions in other legumes, no putative ortholog to *AeCRK* could be found in *M. truncatula* or *L. japonicus*, and actually in no legume using a root hair- and infection-thread mediated infection process (Fig. 7e,f, Supplementary Data 8)."

2. Whether AeCRK is conserved in Papilionoids that have lost nodulation and whether it may also have a non-symbiotic function are good questions. We would like to address these relevant questions in a future study more focused on AeCRK and other symbiotic genes having the same phylogenetic profiles.

3. We followed the suggestion of Reviewer #1 and collaborated with an expert (added as contributor of the present work) in molecular evolutionary analyses. We found that *AeCRK* is subject

to purifying selection in its extracellular domain in the Nod factor-independent *Aeschynomene* clade. This results suggests that *AeCRK* evolution is linked to the acquisition of NF-independent nodulation

Line 333: "To gain further insights into the molecular evolution of *AeCRK*, we ran branch model by estimating different synonymous and nonsynonymous substitution rates (ω=dN/dS) using the phylogenetic tree topology. These analyses, performed on the entire gene sequence and on the four functional domains of *AeCRK* orthologs separately (signal peptide, extracellular, transmembrane and kinase domains), revealed a higher purifying/negative selection acting on the extracellular domain part in the *Aeschynomene* clade (ωBG = 0.480 and ωFG = 0.187, p-val=0.017214) (Fig. 7f , Supplementary Table 24). This purifying selection suggests that *AeCRK* could have evolved to adapt nodulation with *nod* gene-lacking photosynthetic bradyrhizobia."

4. We followed the suggestion of Reviewer #1 and the discussion on *AeCRK* function and evolution was developed:

Line 372: "In line with this idea, a novel receptor-like-kinase belonging to the large CRK family[38] was discovered as being required to trigger nodulation in *A. evenia*. In the legume phylogeny, this gene is present only in Papilionoid lineages using an intercellular infection process and also in the more primitive Caesalpinioid legumes, *Chamaecrista fasciculata* and *Mimosa pudica*, for which infection does not involve root hair invasion either[15]. Such distribution of the *AeCRK* orthologs suggests it could be linked to intercellular infection. Molecular evolutionary analysis further evidenced the extracellular domain of AeCRK orthologs to be under purifying selection in the *Aeschynomene* clade, arguing for a particular evolution with the Nod factor-independent symbiosis. CRKs are repeatedly pointed as important actors of plant early signaling during immunity and abiotic stress[42,43]. They are supposed to be mediators of reactive oxygen species (ROS)/redox sensing through their DUF26 extracellular domains and to transduce the signal intracellularly via their cytoplasmic kinase[43]. Another putative function of their DUF26 domains was recently proposed, based on strong similarity to fungal lectins, as mediating carbohydrate recognition[44]. Therefore, characterization of AeCRK will be crucial to provide information on pending questions: has AeCRK retained the ancestral function or has it been neofunctionalized? Is AeCRK involved in the direct perception of photosynthetic bradyrhizobia or does it mediate ROS/redox sensing during early signaling/infection? Is the Nod factor-independent activation inherently linked to intercellular infection?"

2) Some of the results should be more discussed. The study includes many exciting results that are only shortly discussed. In term of evolutionary processes, the authors could discuss the diversification of Leghemoglobin (same function, different genes). The short parallel with Parasponia could be expanded. More importantly, the authors could elaborate on the impact of their work on deciphering NF-independent nodulation. Does it address some of the current questions? Finding that the entire CSP is present and seems required for nodulation implies that the difference may only come from the receptor plugged-in the pathway. Why are NFP/LYK3 still present (function in AM symbiosis? Other role?).

5. We agree with Reviewer #1 that the discussion was too short and so insufficient to correctly comment all our discoveries. Consequently this section has been completely rewritten and is now well-developped.

6. The expanded discussion now addresses the diversification of Leghemoglobins and the parallel with Parasponia:

Line 407: "Most remarkable is the discovery that Aeschynomene and Arachis spp. have recruited some class 1 Glbs as Lbs transporting O2 in nodule infected cells. Indeed, it is well established that in legumes some class 2 Glbs have evolved to Lbs to ensure such a crucial function36, but the

Dalbergioid lineage appears to be an exception to this pattern of Lb utilization. Comparative genomic analysis in Papilionoid legumes revealed a striking parallel with the presence of two conserved loci where both Glb and Lb genes belonging to class 1 and class 2, respectively, can be found across species. It is therefore tempting to hypothetize that Lbs arose from Glbs by gene tandem duplication and divergent evolution in these two loci, and that they were differentially lost depending on the legume lineages. The presence in the more primitive Caesalpinioid C. fasciculata of a hemoglobin that has some characteristics of Lb37 and is closely related to Dalbergioid Lbs supports that this feature is ancient in legumes. In addition, the presence also in nodulating non-legume species of class 1-derived Lbs (eg. Parasponia) or class 2-derived Lbs (eg. Casuarina) suggests this dual evolution to be recurrent3. This will be an exciting evolutionary issue to determine how different Glbs adapted to Lbs and if these Lbs have any specific functional specificity."

7. The discussion also now elaborates on the impact of our work on deciphering NF-independent nodulation and on symbiotic receptors:

Line 362: "Also striking is the failure of the mutant approach to demonstrate the involvement of any LysM-RLK member, most notably the Nod factor receptors. In agreement with this observation, *LYK3* is not expressed in *A. evenia*. Conversely, *NFP* remains expressed in *A. evenia*, putatively because it has been co-opted from an ancestral role in the arbuscular mycorrhizal (AM) symbiosis in legumes[40]. Therefore, a comparative genetic analysis of *NFP* and *LYK3* between *A. evenia* and *Aeschynomene patula*, which displays a Nod factor-dependent nodulation and which was recently selected as a suitable complementary model[16], should illuminate their recent evolution and clarify if *NFP* has any role in *A. evenia*. Finding that the core Nod signaling pathway, but not the upstream Nod factor receptors, is conserved in *A. evenia* suggests that one main difference with other legumes comes from the symbiotic receptor plugged-in the pathway[41]. In line with this idea, a novel receptor-like-kinase belonging to the large CRK family[38] was discovered as being required to trigger nodulation in *A. evenia*."

Minor comments.

- P7: WDG should be WGD

8. Corrected.

- Reference should be included for the material and method section on Globin spectral analyses

9. Added in the Supplementary Note 2 that contains information on the globin spectral analyses.

Reference: Sainz, M. et al. Plant hemoglobins may be maintained in functional form by reduced flavins in the nuclei, and confer differential tolerance to nitro-oxidative stress. *Plant J.* **76,** 875-887 (2013).

Pierre-Marc Delaux

Reviewer #2 (Remarks to the Author):

The authors de novo assembled a genome for Aeschynomene evenia, which has a unique symbiosis with photosynthetic bradyrhizobia that is Nod factor-independent process. Based on this genome, they did transcriptomic-based assembly for 10 additional Aeschynomene spp. and performed comparative analyses with other legume species that with Nod factor-dependent process. They found numbers of genes that may miss from the Aeschynomene spp. such as LYK3, EPR3, VAG1, SUNERGOS1, BIN3, BIN4…, Also, they screened EMS mutants and identified some previous reported genes and CRK. It is an interesting topic, however, the analyses are not solid enough to support the findings. Here are some comments:

1. Given the high level of sequencing technology, it is easier to get a high quality reference genome now. However, with a scaffold N50 of ~0.985 Mb, the genome assembly of this study is poor. Moreover, only about 302 Mb (80%) of the total length of the assembly were anchored to the chromosomal pseudomolecules. I suggest the authors spend more effort to improve the genome quality since it is not a challenge now. Many approach would help, such as Hi-C and BioNano.

10. We acknowledge the position of Reviewer #2 on our genome assembly. It should be taken into account that the whole project started early 2015 and the genome assembly was launched in parallel to the forward genetic approach. This latter took several years to generate, screen and sequence nodulation mutants. Given the technologies used at that time (PacBio RSII sequencing and scaffold mapping with the use of a genetic map + GBS data), a scaffold N50 of ~0.985 Mb is actually a good result. We do not have the financial and the human capacity to fund Hi-C or BioNano and to perform again the genome assembly, this requirement is too much for the current project. However, we are applying for new fundings as we have the project to exploit a genetic comparative system with *A. evenia* and *A. patula* that uses a Nod factor dependent process. The *A. evenia* genome assembly and annotation will be updated when sequencing and assembling the one of *A. patula* with the use of the newest technologies.

It is important to consider that we obtained very long scaffolds on chromosome arms where the vast majority of protein-coding genes are located. Furthermore, all the analyses performed in this work were correctly done with this genome assembly (synteny with *Arachis* spp., gene search and analysis with no problem of gaps or n blocks). It also supported very successfully the mutant approach to identify symbiotic genes both with the Targeted Sequence Capture and the Mapping-by-Sequencing approaches, further demonstrating the usefulness of the current assembly. Therefore, although we understand Reviewer #2's point of view, we are sincerely convinced that improving the genome quality will yield no meaningful outcome for the genetic study of nodulation in *A. evenia*, which is the real achievement of this work.

As a tentative compromise, we have changed the title of the manuscript to emphasize on the genetics of nodulation and not on the genome:
"Genetics of nodulation in *Aeschynomene evenia* uncovers new mechanisms of the rhizobium-legume symbiosis"
We also shortened and fused the two first parts of the results related to the genome assembly, annotation and analysis (and we did the same with the two figures illustrating the genome structure), while we expanded the other parts of the results dealing with symbiotic genes, based on comments made by the three reviewers. We deeply hope that you will accept that we do not modify the current genome assembly for this publication. Reviewers 1 and 3 did not address such criticism.

2. For the de novo RNA-seq assemblies, how about the linage of the annotation genes from RNA-seq to the annotation of this genome? How many ortholog genes could be identified in the RNA-seq assemblies comparing with the reference genome? The distribution of the gene (coding) lengths? Are they comparable to that from reference genome?

11. For *A. evenia*, we directly aligned Illumina and PacBioIso-Seq sequences to the unmasked genome sequence to facilitate gene annotation (lines 464-467, Supplementary Table 8). No comparison on the gene metrics between the transcriptome and genome assemblies was performed. However, we expect the genome-based transcriptome assembly to be more complete and more reliable than a *de novo* RNAseq assembly. For *A. evenia*, this latter was used for purpose of comparing the number of genes and the BUSCO score with other *Aeschynomene* species for which we developed only RNA-Seq assemblies (Supplementary table 17).

3. For the part of ortholog/comparative analyses part, the procedure is fine, which has been adopted in a number of studies. The authors found some genes may miss from the Aeschynomene spp. like LYK3, EPR3, VAG1, SUNERGOS1, BIN3, BIN4… I would like the authors to add more species, even from species of non-legume to further check if the missing of these genes were indeed associated with Nod factor-dependent/independent. Because missing gene is normal, but if it associate with a specific process is hard to determine, unless clear functional characterization. Now add more species at least to eliminate false judgment.

12. As requested by Reviewer #2, we performed a new OrthoFinder analysis by including more legumes species.

Line 150: "Groups of orthologous genes for *A. evenia*, related *Aeschynomene* spp., and several species belonging to different legume clades were then generated using OrthoFinder (Supplementary Table 18)."

Line 498: "**Orthogroup inference**. We inferred orthogroups with OrthoFinder[57] v.0.4.0 to determine the relationships between *A. evenia*, the other diploid *Aeschynomene* taxa and several legume species. In the latter, proteomes were last obtained from the Legume Information System (https://legumeinfo.org/), the National Center for Biotechnology Center (https://www.ncbi.nlm.nih.gov) or from specific legume species websites in March 2020. They included *A. duranensis* (V14167 v1), *A. hypogaea* (Tifrunner v1), *A. ipaiensis* (K30076 v1), *C. cajan* (pigeonpea ICPL87119 v1), *C. fasciculata* (golden cassia v1), *C. arietanum* (chickpea ICC4958 v2), *L. japonicus* (lotus MG-20 v3), *L. albus* (white lupin v1.0), *L. angustifolius* (narrow-leafed lupin Tanjil_v1.0), *G. max* (soybean Wm82.a2.v2), *M. truncatula* (barrel medic MtrunA17r5.0), *M. pudica* (sensitive plant v1), *P. vulgaris* (common bean G19833 v2) and *V. angularis* (cowpea Gyeongwon v3)."

We did not include non-legume species, because the aim of the study is to search for gene variations linked to the Nod factor-dependent/-independent symbioses within legumes and not to the nodulation/non nodulation traits in plants. However extensive phylogenetic analyses that include legumes and non-legume species can be found for several symbiotic genes in Griesmann et al., 2018.

4. One more, based on the data, it is hard to see that NFP was not detected in all Aeschynomene transcriptomes (Fig. 3a).

13. We took into account the comment and we added red crosses in the empty boxes of the table summarizing the presence/absence of symbiotic genes in legume species. These red crosses make more visible where genes are missing.

5. The EMS database is interesting and would be helpful for further functional analyses. They found several reported genes: AePOLLUX, AeCCaMK, AeCYCLOPS, AeNSP2 and AeNIN. These genes also important for Nod factor-dependent process or even some closely related to Nod factor, does

that mean Nod factor-independent process and Nod factor-dependent process still shared common pathway? Or another explanation?

14. Yes, we do completely agree with this observation. Most of the symbiotic signaling pathway described in model legumes appears to conserved. This point is now addressed in the discussion.

Line 349: "This work leads to the demonstration that the triggering of nodulation in *A. evenia* is mediated by several components of the Nod signaling pathway described in model legumes, *AePOLLUX*, *AeCCaMK*, *AeCYCLOPS*, *AeNSP2*, and *AeNIN*, thus significantly extending a previous report of the involvement of *AeSYMRK*, *AeCCaMK*, and *AeLHK1* genes in root nodulation[13]."

Line 370: "Finding that the core Nod signaling pathway, but not the upstream Nod factor receptors, is conserved in *A. evenia* suggests that one main difference with other legumes comes from the symbiotic receptor plugged-in the pathway[41]."

6. The author found that CRK might be a good candidate for the Nod factor-independent process. Because there is a gene cluster, it might be a little bit complicate. Again, using more species (including species of more legume, non-legume, the10 additional Aeschynomene spp, and even the 12 additional resequenced A. evenia accessions) to check the phylogenetic relationship (instead of current Figure 8e), what is the results could be? I suggest to do more detail analyses in this part. It may make the data clear.

15. As suggested by Reviewer #2, we performed a more complete phylogenetic analysis that includes all the *Aeschynomene* species and additional legume species used in OrthoFinder. These data can be seen in Fig. 7f, and Supplementary Fig.23 and 24. However the 12 additional resequenced *A. evenia* accessions were not included because for these lines, only sequence variations relative to the reference line were searched in the frame of this work. It is also important to note that genes in cluster are often not correctly annotated and therefore such analysis can require a careful genome inspection and manual corrections.

7. One more, the authors find a number of genes that may lost from the Aeschynomene spp. It is interesting that from the comparative, did the authors find any gain genes (beside the additional genes from gene cluster)? If so, when do the mutant screening, if these genes got mutation, does there any phenotype?

16. This is a relevant approach to try to identify genes that account for a considered trait. Such approach was notably used for Parasponia by comparison with the related non-nodulating Trema species (van Velzen *et al*., 2018). We would like to conduct such *in silico* genomic analysis when genomes for Nod-factor dependent *Aeschynomene* species, such as *A. patula*, are sequenced. With the mutant screening, we identified a novel gene resulting from duplication in tandem. This duplication is found only in the Nod factor-independent *Aeschynomene* species. We are currently characterizing this gene and investigating if it has any functional specificity.

Reviewer #3 (Remarks to the Author):

A long-standing objective in legume-rhizobium research is to unravel the molecular genetic pathways controlling the nitrogen-fixing nodule symbiosis, in such that this knowledge can be used to engineer this trait in non-leguminous crops. Nitrogen-fixing nodule symbiosis is not unique to legumes, though current knowledge on the symbiotic gene networks is largely based on studies on two legume species of a single taxonomic clade; Medicago truncatula and Lotus japonicus (both belonging to the Hologalegina clade of the Papilionoideae subfamily). Two decades of research on these models has

provided insights into the genetic pathways essential for establishing this symbiosis. Also, it was shown that these pathways are highly conserved in both species. Despite this substantial body of knowledge, the evolutionary basis of the nitrogen-fixing nodule symbiosis has not been uncovered. Only recently phylogenomic studies using nodulating non-model species provided some insights, indicating the recruitment of three genes to act specifically in the nitrogen-fixing nodule symbiosis. (i) The LysM-type receptor NFP/NFR5 to perceive lipo-chitooligosaccharide signal molecules (known as Nod factors) secreted by the nitrogen-fixing microsymbiont, (ii) the symbiosis responsive transcription factor NIN that is essential -and sufficient- to trigger nodule organogenesis, and (iii) the coiled-coil protein RPG controlling the bacterial infection process.

The Arrighi team now presents a highly relevant system that can be used to obtain novel insights in an alternative evolutionary trajectory of the nitrogen-fixing symbiosis trait; Aeschynomene evenia. A. evenia represents a different taxonomic clade when compared to Medicago / Lotus (Dalbergioids, Papilionoideae). Dalbergioid species have a distinct infection mechanism when compared to most other legumes, not making use of root hair curling-based infection to guide rhizobium to the nodule primordium. Instead, rhizobia penetrate intercellularly via a mechanism known as crack entry. However, the most striking feature of A. evenia is that it is nodulated by rhizobia that do produce the canonical Nod factor signal molecules.

There are two aspects that make this manuscript highly relevant.
(1). The Arrighi team not only has generated a high-quality genome sequence of A. evenia (plus draft genome sequences of 12 additional accessions) but also has integrated it with the genetic map. This facilitates forward genetic screens, as is demonstrated in the manuscript to obtain insights in the molecular genetic mechanisms controlling Nod factor-independent nodulation. This achievement is unique in nodulation research, where forward genetic screens were limited to Medicago and Lotus, and occasionally a legume crop. Therefore, A. evenia can be considered as a third legume model species amenable for genetic research.
(2). By comparative analysis in combination with a close to saturating forward genetic screen, the core symbiosis network for Nod factor-independent nodulation has been identified. A whole set of genes known to be important for nodulation in Medicago and/or Lotus, showed not to be essential for nodulation of A. evenia; including LysM-type Nod factor receptors and the interacting flotillin (FLOT) protein, the LysM-type rhizobium exopolysaccharide receptor EPR3, the chitinase-like enzyme encoding gene NOD FACTOR HYDORLOASE1, RPG, and the genes controlling endoreplication in nodule cells VAG1, SUNERGOS1, BIN3 and BIN4. Additionally, by forward genetics a novel Cysteine-Rich receptor-kinase (AeCRK) was found to be essential for nodule formation in A. evenia. Taken together, I consider these insights highly relevant in the understanding of alternative molecular mechanisms that can lead to nodulation. This knowledge will be instrumental in engineering approaches.

Major issues:

On page 4 it is stated that A. evenia possesses the Nod factor receptor gene NFP, but that this gene is not expressed in a nodulation context. However, when I evaluate the phylogenetic tree presented in Fig. 3B, I do not find a support that A. evenia indeed has a gene orthologous to Medicago MtNFP and Lotus LjNFR5. The closest A. evenia homolog, Ae05g05580, is grouping with MtLYR1/LjLYS11. MtNFP/LjNFR5 and MtLYR1/LjLYS11 evolved most probable upon a Papilionoid-specific whole-genome duplication (see: Young et al., 2011 Nature). MtNFP/LjNFR5 is essential for rhizobium Nod factor-induced nodulation in Medicago and Lotus, however, no evidence has been found that MtLYR1/LjLYS11 plays a role in nodulation as well. Fig. 3B further indicates that also Arachis duranensis lacks a MtNFP/LjNFR5 ortholog, as Aradu.AXP1M also is more closely related to MtLYR1/LjLYS11 than to MtNFP/LjNFR5. These issues have to be resolved. Please, provide irrefutable

evidence that A. evenia (and Arachis species) possesses an MtNFP/LjNFR5 orthologous gene (e.g. by phylogenetic analysis and protein alignments, genomic syntheny conservation, etc).

17. We agree with Reviewer #3 that the tree topology was ambiguous to show the orthologous relationship of Ae05g05580 with MtNFP and LjNFR5. To address this issue, we used another phylogenetic method as indicated line 521. Now this orthologous relationship is unequivocal (see Fig. 2b). We also present a detailed synteny analysis showing clearly that the genomic region containing Ae05g05580 in *A. evenia* is closest to the one containing MtNFP than the one having MtLYR1 in Medicago (Supplementary Fig. 7a).

No complementation has been conducted for AeCRK, and I am wondering why, as Agrobacterium rhizogenes mediated transformation has been established for A. evenia (Arrighi et al 2012 MPMI). To my opinion, complementation of the Aecrk mutant is an omission.

18. We agree with Reviewer #3 that a functional complementation is missing for AeCRK. We performed an *A. rhizogenes* mediated transformation of an Aecrk mutant with a construct containing the AeCRK cDNA fused to its native promoter. This construct successfully restored nodulation in the mutant plants as explained in the results:

Line 320: "Hairy root transformation of the I10 mutant with the coding sequence of *AeCRK*, fused to its native promoter, resulted in the development of nodules upon inoculation with the *Bradyrhizobium* ORS278 strain, while no nodules were produced in control plants transformed with the empty vector (Supplementary Fig. 23, Supplementary Table 23). The identification of genetic lesions in the two independent *Aecrk* alleles together with the transgenic complementation of the mutant phenotype provide unequivocal evidence that *AeCRK* is required for the establishment of symbiosis *A. evenia*."

It is reported that Aecrk mutants are nod minus, however, no detailed phenotypic analysis is presented. So, can it be confirmed that nodule organogenesis is fully blocked in Aecrk mutant plants? And what about rhizobium-induced NIN expression? Some basic phenotypic characterization is welcomed.

19. We do confirm that no nodule develop on the Aecrk mutants. In several congresses, we have presented for *A. evenia* other mutants able to occasionally develop WT nodules, this is not the case for the two Aecrk mutants. We understand Reviewer #3's wish to have more data presented for this gene. However, investigating in detail the role of (conserved and novel) symbiotic signaling genes in a Nod factor- and infection thread-independent symbiosis deserves to constitute another story. Currently, we are conducting a detailed phenotypic and molecular (Q-PCR) characterization of the Nod- mutants altered in the different signaling genes. Our aim is to compare their phenotypes to the one described in model legumes for the mutants mutated in orthologous genes and to position AeCRK relative to other genes of the symbiotic signaling pathway.

Gene loss in A. evenia is described for EPR3, NFH1, RPG, FLOT1, VAG1, SUNERGOS1, BIN3, BIN4. However, the underlying data are not always obvious. Are the genes not annotated, pseudogenezed, completely eroded/deleted? Please, provide the data (as supplemental information) that unambiguously support the absence of functional copies of these genes.

20. Data were clarified in the text as requested and more detailed synteny analysis are provided as supplemental Information. Gene loss or alteration was investigated based on gene annotation, direct Blast on the genome and synteny analysis in CoGe (allows a genomic sequence comparison (that is not dependent on gene annotation). A complete absence for EPR3, NFH1, FLOT, SUNERGOS1, BIN3, BIN4 and a partial deletion for VAG1 was observed. RPG was not investigated because this

genes is absent in Arachis species that belong to the same Dalbergioid clade, indicating that this loss is not specific to the Aeschynomene lineage.

Line 165: "In line with this observation, the gene coding for NFH1 (Nod Factor Hydrolase 1), which mediates Nod factor degradation in *M. truncatula*, was not found in any *Aeschynomene* data (Fig. 2a and Supplementary Data 5). Interestingly, *EPR3*, which inhibits infection of rhizobia with incompatible exopolysaccharides in *L. japonicus*, was not found in the *A. evenia* genome (Fig. 2a). Synteny analysis based on genomic sequence comparison with *A. duranensis* confirmed the complete deletion of *EPR3*, of genes within the *LYK* cluster containing *LYK3* and of the *NHF1* gene in *A. evenia* (Supplementary Fig. 7-9)."

Line 192: "*RPG* was undetectable in Dalbergioid legume species and *FLOT* genes were completely missing in *Aeschynomene* spp."

Line 205 : "Synteny analysis based on genomic sequence comparison with *Arachis* spp. substantiated the specific and complete loss of *SUNERGOS1*, *BIN3*, and *BIN4*, and the partial deletion of *VAG1* in *A. evenia* (Supplementary Fig. 13)."

page 8: Provide a list -supported by phylogenetic analysis- which LysM-RLK genes are absent in A. evenia.

21. Done in Supplementary Data 4. Missing genes are indicated by a "-" in the table.

Minor issues:

The loss of RPG is not unique to Aeschynomene, but also has been lost in Arachis. As RPG (like NFP) is considered to be one of the three genes that specifically evolved allowing nodulation, this finding should be addressed in the DISCUSSION section of the manuscript.

22. We added a topic on RPG in the discussion:

Line 391: "the ability of *L. japonicus* to be infected intracellularly or intercellularly, depending on the rhizobial partner, was recently used to provide insights into the genetic requirements of intercellular infection[46]. It was showed that some determinants required for the infection thread-mediated infection are dispensable for intercellular infection, among which RPG is found. This finding echoes the observed absence of RPG in *A. evenia* and other Dalbergioid legumes for which intercellular infection is the rule. However, other infection determinants (LIN, VPY, EXO70H4 and SYN) that are also involved in intracellular accommodation of symbionts are present in *A. evenia*, suggesting that both the core symbiotic signaling pathway and the machinery mediating intracellular accommodation are conserved, as a general feature of endosymbioses[47]."

As several mutant alleles for POLLUX, CCAMK, CYCLOPS, NSP2 and NIN have been found, I consider your screen for nod minus mutants close to saturation. To my surprise, no NSP1 mutants have been found, whereas there are no obvious paralogs reported that may function redundantly (like is the case for SYMRK). This should be addressed in the DISCUSSION section of the manuscript.

23. The absence of nsp1 mutants is now addressed in the discussion:

Line 356: "With the forward genetic screen, not all known genes of the Nod signaling pathway were recovered. Indeed, no causative mutation could be found in *AeCASTOR* or in *AeNSP1*, whereas CASTOR and NSP1 are known to act in concert with POLLUX and NSP2, respectively[1]. In addition,

there are no obvious paralogs reported that may function redundantly, as it is probably the case for SYMRK in *A. evenia*. Therefore, either both these genes were unfortunately not targeted by the EMS mutagenesis or a special evolution of *AePOLLUX* and *AeNSP2* rendered them sufficient for symbiosis as evidenced for DMI1/POLLUX in *M. truncatula*[39]."

page 4: "Previous transcriptome analysis did not detect the expression of several known genes involved in bacterial recognition, infection and nodule functioning". Provide information on which genes have been studied previously, and what tissues have been used for this.

24. We modified the text to indicate which genes have been studied previously, and what tissues have been used for this:

Line 88: "Previous transcriptomic analysis from root and nodule tissues did not detect expression of several known genes involved in bacterial recognition (e.g. *LYK3* and *EPR3*), infection (e.g. *RPG* and *FLOT*) and nodule functioning (e.g. *SUNERGOS1* and *VAG1)*[12,18]. Such data support the presence of new or divergent symbiotic mechanisms in *A. evenia* in comparison with other well-studied model legumes."

page 6: Provide web address for AeschynomeneBase.

25. The web address is now provided.

Line 129: "all the resources were incorporated in the AeschynomeneBase (http://aeschynomenebase.fr)"

page 7, last paragraph. Fig. 3A is only a schematic representation of the gene species tree. Please, provide a high-resolution consensus species tree based on the inference of single copy orthogroups.

26. We provide the consensus species tree inferred from single-copy orthogroups by OrthoFinder. As indicated in the legend of Fig. 2 (line 805), all BS values (x1000) were comprised between 92 and 100% and so are not indicated for figure clarity.

page 15, DISCUSSION, first paragraph. Information on "T3SS-mediated hijacking of the symbiotic signalling pathway" and " the absence of the T3SS in A. evenia compatible photosynthetic Bradyrhizobium species" has to be included in the INTRODUCTION of the manuscript.

27. Done

Line 78: "In this case, nodulation is not triggered by a hijacking Type-3 secretion system present in some non-photosynthetic bradyrhizobia[9,10]"

Fig. 3A: The use of a single letter genus abbreviation for Arachis and Aeschynomene is confusing. Can be solved by using either two letters (Ar and Ae) or a colour indication of Aeschynonomene.

28. As suggested, to better distinguish *Aeschynomene* from *Arachis* species, the name of *Aeschynomene* species are now in red in Fig. 2 and 3.

Fig. 3A and Fig. 4A can be merged. Fig. 4B could go to the supplement.

29. We agree that Fig. 3a and 4a are similar, but as they refer to different early and late steps of nodulation, respectively, we would prefer to maintain them separate. Also, Fig. 3a and 3b show an opposed correlation between the presence or not of genes, and ploidy levels in nodules.

Fig. 3C: Include also EPR. To create space, Fig. 3D can go to the supplement.

30. Done: EPR3 is now clearly indicated in Fig. 2C, and Fig. 2D has moved to the supplement as Supplementary Figure 6.

Fig. 8f: Provide gene IDs of CRK genes (black arrows) similar as in Fig. 6d (LB genes).

31. As we needed more space on the figure to comply with Reviewer #3's request, we moved the microsynteny analysis to the supplement as Supplementary Figure 24 and added gene IDs of CRK genes.

Suppl. Fig. 4a, Supple Fig. 5a-b: Provide gene IDs of LYK, SYMRK, and PUB1 genes (black arrows in figures) similar as in Fig. 6d (LB genes).

32. Done as requested for all these genes.

Suppl. Fig. 4b-c, Suppl. Fig. 5c, Suppl. Fig. 6a-d: I find these screenshots messy and not easy to interpret. Please, replace them by schematic drawings (e.g. as in Suppl. Fig. 4a and Suppl. Fig. 5a-b) and provide all the alignments in a separate supplemental file.

33. We replaced all the screenshots by schematic drawings. Presentations of the synteny analyses are clearer and homogeneous now. We also expanded the number of supplementary figures so that the drawings are not stacked anymore and are easier to observe.

Reviewer #1 (Remarks to the Author):

The edited version of Quilbé et al. addressed most of my previous comments. Beside my own comments, I appreciated to see the complementation assays of the Aecrk mutant which was missing from the previous manuscript. At this stage I do not have technical/methodological concerns and I am satisfied with the presented work.

However, I would like the author to edit the text:

- “primitive” (L325, L416) is incorrectly used. There is no reason to think that any of the extant legume clades are more primitive than the others. Please rephrase. Occurrence of AeCRK in Mimosa and Chamaecrista and absence in most Papilionoid (thus the paraphyletic occurrence) suggests that the presence is ancestral in legumes.

- “Conversely, NFP remains expressed in A. evenia, putatively because it has been co-opted from an ancestral role in the arbuscular mycorrhizal (AM) symbiosis in legumes40.” I think this sentence is misleading. The gene might be maintained because of a function in AM symbiosis, which is likely ancestral (see Girardin et al. 2019). Please rephrase.

I congratulate the authors for their great work.

Pierre-Marc Delaux

Reviewer #2 (Remarks to the Author):

I am sorry that I did not see too much improvement from the revised manuscript. Most of my concerns are not addressed.

[Editor: Reviewer #3 states in Remark to Editor section that (s)he supports the publication of this manuscript.]

REVIEWERS' COMMENTS

Reviewer #1 (Remarks to the Author):

The edited version of Quilbé et al. addressed most of my previous comments. Beside my own comments, I appreciated to see the complementation assays of the Aecrk mutant which was missing from the previous manuscript. At this stage I do not have technical/methodological concerns and I am satisfied with the presented work.

However, I would like the author to edit the text:

- "primitive" (L325, L416) is incorrectly used. There is no reason to think that any of the extant legume clades are more primitive than the others. Please rephrase. Occurrence of AeCRK in Mimosa and Chamaecrista and absence in most Papilionoid (thus the paraphyletic occurrence) suggests that the presence is ancestral in legumes.

1. **The word "primitive" was removed from the text (L371 and L410) and the sentence (L371) in the discussion was rephrased as requested.**

- "Conversely, NFP remains expressed in A. evenia, putatively because it has been co-opted from an ancestral role in the arbuscular mycorrhizal (AM) symbiosis in legumes40." I think this sentence is misleading. The gene might be maintained because of a function in AM symbiosis, which is likely ancestral (see Girardin et al. 2019). Please rephrase.

2. **L361: the sentence was rephrased as resquested.**

I congratulate the authors for their great work.

Pierre-Marc Delaux

Reviewer #2 (Remarks to the Author):

I am sorry that I did not see too much improvement from the revised manuscript. Most of my concerns are not addressed.

[Editor: Reviewer #3 states in Remark to Editor section that (s)he supports the publication of this manuscript.]
